# mOTUpan: a robust Bayesian approach to leverage metagenome-assembled genomes for core-genome estimation

**DOI:** 10.1093/nargab/lqac060

**Published:** 2022-08-15

**Authors:** Moritz Buck, Maliheh Mehrshad, Stefan Bertilsson

**Affiliations:** Department of Aquatic Sciences and Assessment, Swedish University of Agricultural Sciences, Lennart Hjelms väg 9, 75651 Uppsala, Sweden; Department of Aquatic Sciences and Assessment, Swedish University of Agricultural Sciences, Lennart Hjelms väg 9, 75651 Uppsala, Sweden; Department of Aquatic Sciences and Assessment, Swedish University of Agricultural Sciences, Lennart Hjelms väg 9, 75651 Uppsala, Sweden

## Abstract

Recent advances in sequencing and bioinformatics have expanded the tree of life by providing genomes for uncultured environmentally relevant clades, either through metagenome-assembled genomes or through single-cell genomes. While this expanded diversity can provide novel insights into microbial population structure, most tools available for core-genome estimation are sensitive to genome completeness. Consequently, a major portion of the huge phylogenetic diversity uncovered by environmental genomic approaches remains excluded from such analyses. We present mOTUpan, a novel iterative Bayesian method for computing the core genome for sets of genomes of highly diverse completeness range. The likelihood for each gene cluster to belong to core or accessory genome is estimated by computing the probability of its presence/absence pattern in the target genome set. The core-genome prediction is computationally efficient and can be scaled up to thousands of genomes. It has shown comparable estimates to state-of-the-art tools Roary and PPanGGOLiN for high-quality genomes and is capable of using genomes at lower completeness thresholds. mOTUpan wraps a bootstrapping procedure to estimate the quality of a specific core-genome prediction, as the accuracy of each run will depend on the specific completeness distribution and the number of genomes in the dataset under scrutiny. mOTUpan is implemented in the mOTUlizer software package, and available at github.com/moritzbuck/mOTUlizer, under GPL 3.0 license.

## INTRODUCTION

The continuous advancements of high-throughput sequencing technologies and bioinformatics tools over the last two decades have fueled large-scale ecogenomic analyses leading up to a new view of the tree of life (1–3). This refined view enabled by metagenomics and single-cell genomics reveals that uncultured bacteria and archaea exclusively represented by metagenome-assembled genomes (MAGs) and single-cell amplified genomes (SAGs) account for ∼75% of the cataloged phylogenetic microbial diversity (2). Despite their unequivocal potential to reveal diversity, the inherent incompleteness of MAGs and SAGs has so far hindered attempts in the large-scale study of subpopulation diversity, core-genome structure and genome evolution of these phylogenetically diverse species.

All nonredundant genes in genomes from a genome set are part of its pan-genome and can be categorized as either core or accessory (4). The core genome is a set of genes common among all genomes of a species and is supposedly responsible for the basic aspects of the cell’s biology and phenotypic traits (5). The accessory part of the genome is underpinning the subspecies diversity and is defined as genes present in two or more but not all representatives of a species. Accessory genes typically encode for functions that provide cells with adaptive advantages (e.g. supplementary metabolic pathways, enzymatic activities, antibiotic resistance, phage and predation resistance, pathogenicity, etc.) (4–6), but are often also relics or live selfish genetic elements (7).

A key prerequisite for the comparative analyses of the subspecies diversity and ecological adaptations is to first have a robust estimation of the core genome that will enable a better assessment of the accessory counterparts. However, core-genome analyses are limited in taxonomic scope (8–13), largely because of the severe limitations in culturing microbes and obtaining high-quality genomes, combined with existing bioinformatics methods being dependent on high-quality genomes to scaffold such analyses. Most methods used for core-genome analysis only work with sets of high-quality and complete genomes and are very sensitive to missing genes and fragmented genomes (14). These methods often concentrate on developing novel methods for computation of clusters of orthologous genes (COGs) in the population of interest (14) and use only simple binary presence/absence models for the core-genome estimation (e.g. a COG is core if it is present in all the genomes of the clade). Such methods perform best when used on a moderate number of high-quality genomes generated from cultured microbial isolates. Accordingly, these methods are unable to deal with the rapidly growing database of incomplete and fragmented MAGs and SAGs of the uncultured majority of Earth’s microbiome (2). Due to these methodological limitations, our understanding of the size and structure of microbial core genomes and pan-genome dynamics remains elusive and lags behind our growing appreciation of microbial phylogenetic diversity. The recently released software, PPanGGOLiN, uses synteny networks to compute clusters of co-occurring gene clusters instead of presence/absence. This method is highly scalable, fast and robust enough to deal with incomplete genomes (15). However, this method could be sensitive to fragmentation, which is a prominent feature of most incomplete MAGs and SAGs, and is not explicitly tailored to find the core, but rather to find clusters of syntenic genes.

Here, we present a novel approach for computing core genomes relying on a Bayesian estimator of the observed presence/absence patterns of discrete genome-encoded traits (any trait that can be encoded in a genome, e.g. gene cluster, COG, functional annotations, etc.) in sets of incomplete MAGs/SAGs and complete genomes. We wrote a software tool, mOTUpan, that can estimate whether any genome-encoded trait is more likely to be present in all genomes of a genome set or only in a subset. mOTUpan can compute the core-genome partitioning for genome sets of a wide range of qualities, and is computationally efficient, agnostic to the genome-encoded traits used and very robust to incompleteness.

## MATERIALS AND METHODS

### Bayesian approach for core-genome estimation

mOTUpan can use any set of genomes that is suspected to share a certain number of genome-encoded traits. We typically use clusters where all genomes are within compact clusters defined by a 95% average nucleotide identity (ANI) threshold. We call such clusters metagenomic operational taxonomic units (mOTUs), which can be seen as an operational definition of species. However, genomes clustered at any other taxonomic level, or any other way one can imagine (by niche, predator, host, etc.), could be done too, but one should consider turning off re-estimation of completeness estimates in some cases (‘--max_iter 1’). We will use the term genome as a shorthand for any set of nucleotide sequences originating from the same organism. This could be draft genomes, complete genomes, MAGs or SAGs. Each genome is first described as a set of genome-encoded traits. Here, we will use gene clusters, but it should be mentioned that mOTUpan is agnostic to the specific form of such traits; one could use genes, COGs, functional annotations or any other discrete trait that is encoded by a genome. mOTUpan then uses an iterative Bayesian approach to classify each trait of the genome in a genome cluster as a core or accessory trait based on a likelihood ratio. For each of the two hypotheses (core or accessory trait), a probability is computed using an initial genome completeness estimate inferred for each genome [genome completeness can be calculated using CheckM (16) or any other tool of your choosing, or a fixed value used]. The most likely trait category (core or accessory) is then picked as class for that trait. Using this new classification, we re-estimate completeness, which can be used as an estimate for a second iteration and then repeat this entire process until convergence.

### Probability models

To compute the probability of a distribution of a specific trait in the genome set mOTU under the assumption that it is in the core, we multiply the probability *p*_trait∈*g*|core_ of any genome *g* (*g* is treated as a set of traits) that has that gene cluster with the inverse probability 1 − *p*_trait∈*g*|core_ for the genomes that do not have that trait, where the probability *p*_trait∈*g*|core_ is actually directly the completeness estimate *c*_*g*_ of *g*, e.g. Equations (1) and (2):

(1) $$\begin{equation*} p_{{\rm trait}|{\rm core}}=\prod _{\substack{{g} \in {\rm mOTU}\\ {\rm if\,trait} \in {g}}} p_{ {{\rm trait}} \in {{g}|{\rm core}}} \prod _{\substack{ {g} \in {\rm mOTU}\\ {\rm if\,trait} \notin {g}}}(1-p_{{{\rm trait}} \in {g|{\rm core}}}), \end{equation*}$$

(2) $$\begin{equation*} p_{{\rm trait} \in {g|{\rm core}}}=c_g .\end{equation*}$$

For the probability under the assumption that it is in the accessory fraction of the genome, we will have to make some further assumptions with regard to the structure of the pan-genome. We have assumed that the traits in the pan-genome that are not in the core are independent, and each trait has a frequency ${|{\rm trait}|}/{|T|}$, where |trait| is the number of genomes in mOTU that have that trait and |*T*| is the total size of the traits’ pool, e.g. $\sum _{\substack{{\rm all traits}}}{|{\rm trait}|}$. To ‘fill’ the accessory fraction of a genome, we draw ‘|*g*|’ times, where |*g*| is the number of traits in the genome, core size |core_mOTU_| and completeness *c*_*g*_, resulting in Equations (3) and (4):

(3) $$\begin{eqnarray*} p_{{{\rm trait}|{\rm access}}}&=&\prod _{\substack{ {g} \in {\rm mOTU}\\ {\rm if\,trait} \in {g}}} (1-\overline{p}_{{\rm trait} \in {g|{\rm access}}}) \prod _{\substack{ {g} \in {\rm mOTU}\\ {\rm if\,trait} \notin {g}}} \overline{p}_{{\rm trait} \in {g|{\rm access}}} ,\nonumber\\ \end{eqnarray*}$$

(4) $$\begin{equation*} \overline{p}_{{\rm trait} \in {g|{\rm access}}}=\left(1-\frac{|{\rm trait}|}{|T|}\right)^{|g|-c_g|{\rm core}_{{\rm mOTU}}|} .\end{equation*}$$

For practical reasons, these computations are all done in log space, resulting in a log-likelihood ratio (LLHR)

(5) $$\begin{equation*} {\rm LLHR}=\log {(p_{ {{\rm trait}| {\rm core}}})}-\log {(p_{ {{\rm trait}|{\rm access}}})} .\end{equation*}$$

If the LLHR of Equation (5) is positive, the trait is considered core; if negative, it is considered accessory. Using this classification, we recompute an updated completeness estimate for each genome:

(6) $$\begin{equation*} c_g=\frac{|{\rm core}_{\rm mOTU} \cap {g} |}{ | {\rm core}_{\rm mOTU}|}, \end{equation*}$$

where core_mOTU_ is the set of all traits classified as core.

After this step, we rerun the likelihood computation. This is repeated until convergence (when core-genome estimates remain unchanged), to obtain a final set of core traits and accessory traits, and posterior completeness estimates.

### Benchmarking mOTUpan for core-genome estimation

To benchmark the core genomes computed by mOTUpan against other commonly used core-genome analysis tools, we calculated the core genomes for 301 species containing a total of 11570 genomes (for larger species, only 50 genomes were randomly picked to make the runs tractable with Roary) from the Genome Taxonomy Database (GTDB release 95) (3) and 258 mOTUs containing 8955 genomes in total from the StratFreshDB (17). The MAGs were reclustered with mOTUlizer (github.com/moritzbuck/mOTUlizer), which computes a network based on average nucleotide identity of which the connected components form OTU-like clusters [see (17) for more details], with less stringent parameters (‘--MAG-completeness 30 --MAG-contamination 10’) to have more low-quality mOTUs and compare the performance of mOTUpan to Roary (14) (version 3.13.0) and PPanGGOLiN (15) (version 1.1.96). Normalized residues of the comparisons are computed by dividing the difference between mOTUpan’s predicted core size and Roary/PanGGOLiN’s predicted core size by the mean of the predictions. Genome statistics, accession numbers and taxonomy are available in Supplementary Table S1. This step aims to highlight and compare the performance of mOTUpan with Roary and PPanGGOLiN with regard to the ability to handle incomplete and fragmented genomes.

For more detailed benchmarking of mOTUpan performance, we selected a dataset of genomes affiliated with the *Prochlorococcus_A* genus from the GTDB. All genomes classified as *Prochlorococcus_A* according to GTDB-Tk (18) found in RefSeq as well as GORG (19) were clustered into mOTUs (using mOTUlizer with standard parameters); the mOTU with the largest number of genomes was used (see Supplementary Table S2 for genome statistics and accession numbers). This *Prochlorococcus* mOTU consists of 388 genomes whereof 3 are closed genomes and 16 genomes are estimated to be >95% complete according to CheckM (16) (version 1.1.3) results. Genomes assigned to this mOTU range in completeness from 8.59% to 99.52% (median = 69.05%) (Supplementary Table S2). mOTUpan’s performance for core-genome estimates for this *Prochlorococcus* mOTU was benchmarked against PPanGGOLiN using the gene clusters generated by it [PPanGGOLiN uses mmseqs (20) internally for gene clustering, version 13.45111 in our case]. All results shown in this paper were analyzed by mOTUpan version 0.3.2.

### Bootstrapped false discovery rate and sensitivity

In addition to the likelihood ratio between the two probabilities, a bootstrapping approach has been integrated in mOTUpan to estimate the false discovery rate and sensitivity of a specific partitioning. Synthetic genomes are built by drawing gene clusters from the original genome set according to the partitioning; e.g. every synthetic genome is composed of all the core gene clusters, and a number of accessory gene clusters are drawn randomly from the pool of accessory gene clusters based on an estimated genome size (mean of number of gene clusters divided by completeness estimate). The synthetic genomes are built ‘complete’ and then rarefied by randomly removing gene clusters according to the genome set’s posterior completeness estimates. This synthetic set of genomes is then run through mOTUpan again and the counts of core traits in the obtained core genome and accessory are used to estimate the false positive rate and sensitivity. Multiple synthetic datasets can be analyzed to obtain a better estimate. To evaluate the bootstrapping, we need a core genome that is assumed to be true. To achieve this, we ran 10 runs of mOTUpan with 100 randomly picked genomes of *Prochlorococcus_A* selected from the set used for benchmarking. We used the union of the obtained cores as such (this is a liberal estimation of the true core as we cannot know what the true core of this population is). We then for each run in the bootstrapping computed an empirical false positive rate by counting the genes appearing in the computed core that are not a part of our calculated true core from the previous step. We then end computed a bootstrapped false positive rate. Results are presented in Supplementary Table S3 and Supplementary Figure S1.

## RESULTS AND DISCUSSION

### Overview of the mOTUpan’s Bayesian approach

The Bayesian approach adopted in this tool tries to leverage the genomic diversity uncovered by incomplete and fragmented MAGs and SAGs for exploring the core-genome and pan-genome structure of bacterial and archaeal species (or any other set of genomic traits). Most available tools such as Roary rely on a hard presence/absence threshold for defining the core genome. This limitation renders such tools largely unusable when dealing with incomplete and fragmented MAGs and SAGs. Comparing the performance of Roary and mOTUpan for core-genome estimation with the gene clusters computed by Roary is equivalent to comparing mOTUpan to a hard threshold approach.

The network nature of PPanGGOLiN makes it relatively robust to deal with some degree of incompleteness; however, as it is looking for patterns of synteny to determine the persistent fraction of the genomes, too much fragmentation (that is common in MAGs and SAGs) could cause problems in calculations of the persistent fraction of the genomes. The Bayesian approach of mOTUpan, on the other hand, helps by potentially bypassing both incompleteness and fragmentation limitations for core-genome and pan-genome estimation for sets of incomplete and fragmented MAGs and SAGs. To give an approximation of the runtime and memory usage, we have used 9443 *Staphylococcus aureus* genomes downloaded from the GTDB. These genomes were processed in 4 min for gene clustering on 24 threads by mmseqs2, and 2 h 15 min for mOTUpan on a single thread on a Ryzen 9 3900X using around 3 GB of RAM. mOTUpan also calculates bootstrapped false discovery rate and sensitivity for the core-genome/pan-genome partitioning.

There are widespread and valid concerns that MAGs are contaminated by contigs that might not be a genuine part of their genome, as binning tools may mistakenly cluster them together with the rest of the MAG. MAGs are usually screened for putative contamination with tools such as CheckM that relies on a limited dataset of high-quality genomes to compute a set of markers. mOTUpan can, however, address this known problem in a different way, as genes annotated as core have a very low likelihood of being contaminants and can thus be used for prediction of genome quality. Thus, mOTUpan allows users to compute an alternative to the completeness values estimated by CheckM (or other tools) independent of marker gene collections or complete genomes. This alternative can be used for all kinds of genome sets, such as viruses or plasmids, that do not have dedicated tools or databases.

### Benchmarking mOTUpan against Roary and PPanGGOLiN along the completeness scale

**Figure 1.**
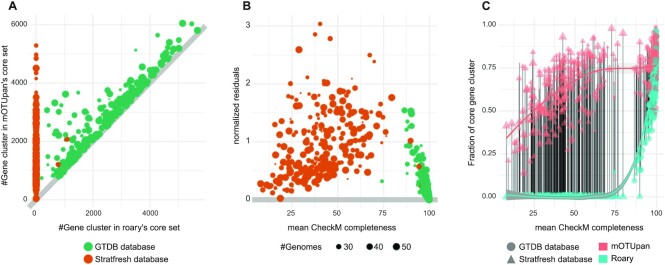
Benchmarking the performance of mOTUpan against Roary along the completeness scale. Three hundred one species containing 11 570 genomes from the GTDB and 258 mOTUs containing 8955 genomes in total from the StratFreshDB are used for this comparison. Gene clusters used are the ones computed by Roary. (**A**) Predicted core sizes. (**B**) Normalized residues, fold change between core size predicted by mOTUpan and Roary; if the number is >1, mOTUpan’s prediction is larger. (**C**) Predicted gene clusters in core divided by estimated number of gene clusters per genome (bins below 40% completeness are ignored for this estimate) versus the mean. Local polynomial regression fitting is used in panel (C).

To benchmark the performance of mOTUpan against Roary, we used the gene clusters generated by Roary. Comparing the performance along the completeness scale shows that Roary is highly sensitive to genome completeness, as Roary’s core-genome estimate drops away considerably from that of mOTUpan when completeness decreases (Figure 1A and B). Some of these limitations can be bypassed by manually adjusting thresholds in Roary, but while this can be done at a small scale, it is not tractable for the larger scales where mOTUpan can still function (as is stated on its web page: ‘Roary is not intended for metagenomics or for comparing extremely diverse sets of genomes’, https://sanger-pathogens.github.io/Roary/).

**Figure 2.**
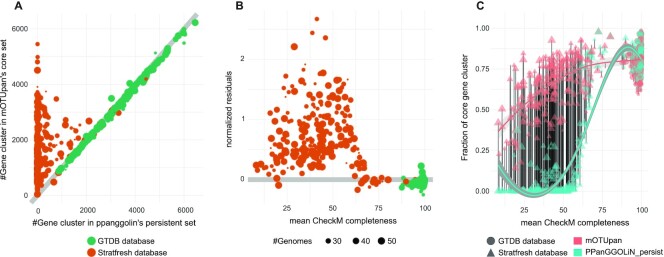
Benchmarking the performance of mOTUpan against PPanGGOLiN along the completeness scale. Three hundred one mOTUs containing 11 570 genomes from the GTDB and 258 mOTUs containing 8955 genomes in total from the StratFreshDB were used for this comparison. Gene clusters used are the ones computed by PPanGGOLiN (based on mmseqs2). (**A**) Predicted core sizes. (**B**) Normalized residues, fold change between core size predicted by mOTUpan and PPanGGOLiN; if the number is >1, mOTUpan’s prediction is larger. (**C**) Predicted gene clusters in core divided by estimated number of gene clusters per genome (bins below 40% completeness are ignored for this estimate). Local polynomial regression fitting is used in panel (C).

Running mOTUpan using the COGs generated by PPanGGOLiN [which internally uses the mmseq2 (15) clustering tool], we obtain similar core-genome estimates for the GTDB dataset (the more complete genome sets) (Figure 2A). Looking more specifically at the deviation from the first bisector along the completeness scale (Figure 2B), we can see that in general PPanGGOLiN’s core-genome estimates are larger than those obtained with mOTUpan for the more complete genome sets. This tendency changes drastically once the average completeness drops below 70% where the mOTUpan estimates become larger. This increase could be due to an inflation of predicted core gene clusters for the more incomplete genome sets. We accounted for this possibility by inspecting the fraction of the genome classified as core (Figure 2C). While this estimate is expected to be independent of completeness, we can see that outputs from both PPanGGOLiN and mOTUpan drop away from the expected value with lower completeness, but the output from PPanGGOLiN drops faster, demonstrating mOTUpan’s robustness to incomplete and noisy genomes. Additionally, PPanGGOLiN is designed to classify genes in three partitions (persistent, shell and cloud) and thus it is not adapted for very incomplete genome sets. While at higher completeness values both tools offer good estimation of the core genome, for most ecological studies that focus on MAGs with completeness at the ≥50% completeness and ≤5% contaminations (2), mOTUpan could provide a better estimation of the core genome.

### Benchmarking mOTUpan against PPanGGOLiN for a *Prochlorococcus_A* genome set

For a more detailed benchmarking of mOTUpan against PPanGGOLiN, we used a set of 388 genomes from the *Prochlorococcus_A* genus, ranging in completeness from 8.59% to 99.52% (median = 69.05%) according to CheckM (Supplementary Table S2). For this analysis, we used the gene clusters generated by PPanGGOLiN.

**Figure 3.**
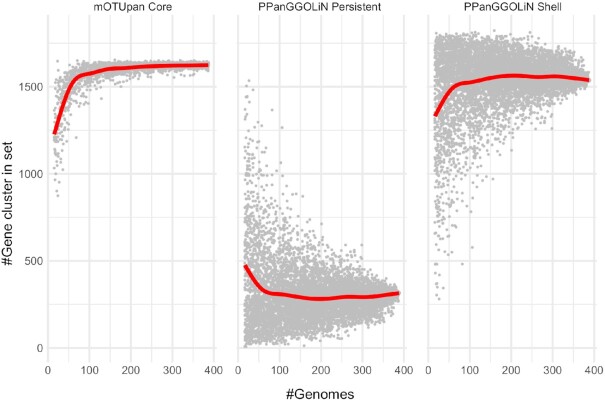
Rarefaction analysis of mOTUpan’s and PPanGGOLiN’s core-genome prediction on the *Prochlorococcus_A* mOTU. The same analysis was performed on random subsets of the available 388 genomes.

**Figure 4.**
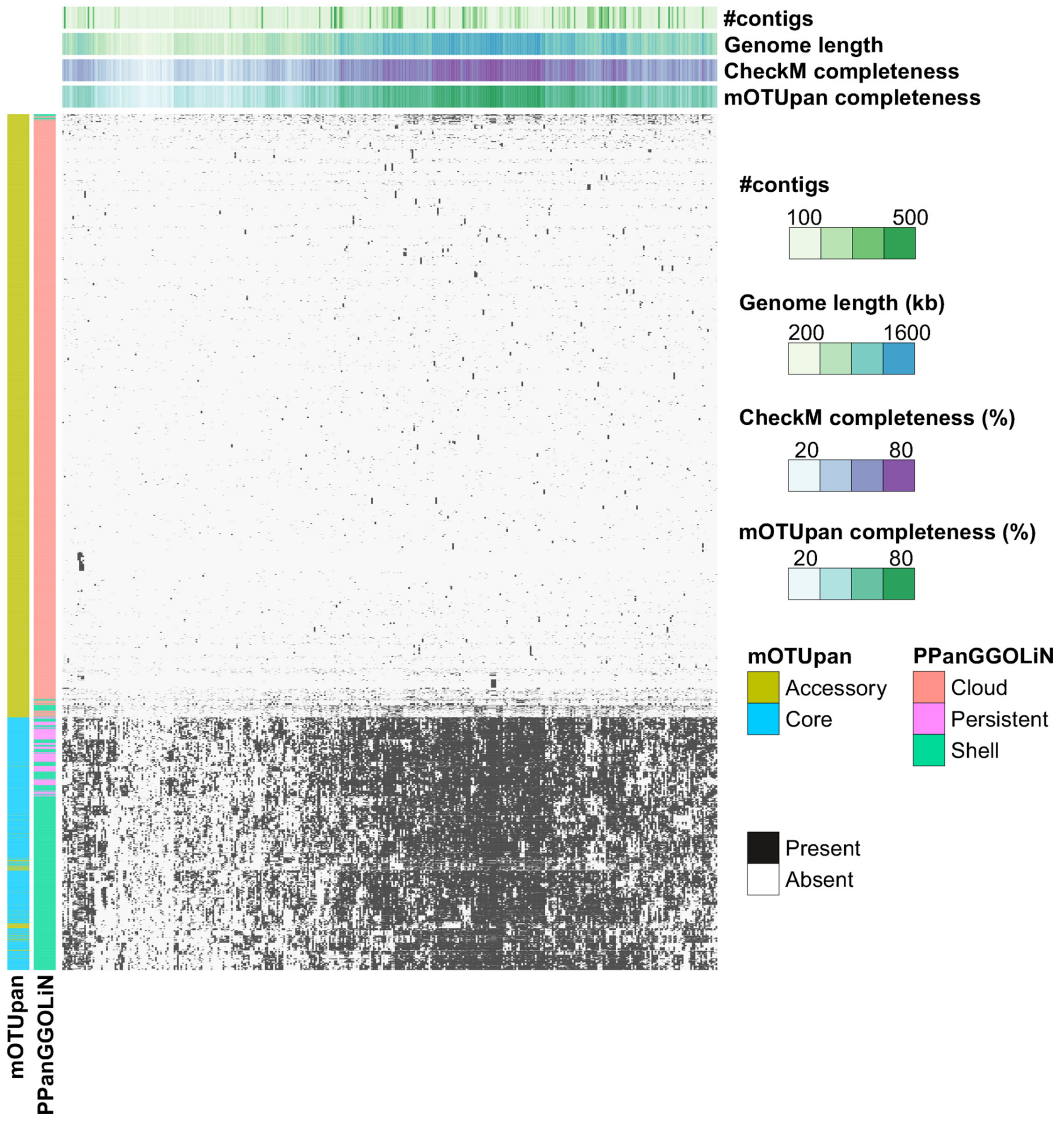
Distribution of 5985 generated gene clusters from 388 genomes of a *Prochlorococcus_A* mOTU. Each column represents a genome, and each row represents a gene cluster. Presence/absence pattern of each gene cluster in each genome is shown in black and white, respectively. Gene clusters are assigned to different partitions using mOTUpan and PPanGGOLiN estimations. These assignments are shown in the left side of the heatmap as colored columns. Genome stats such as number of contigs, genome length, CheckM completeness and mOTUpan completeness are shown on top of the heatmap.

PPanGGOLiN splits the set of gene clusters by default into three subsets: persistent, shell and cloud. For very complete genomes, the persistent set of gene clusters is close to the core genome, but for more noisy genomes, such as those included in this *Prochlorococcus_A* genome set, the approach is not capturing the entire core genome (Figure 3). It is notable that gene clusters identified as ‘persistent’ (316 gene clusters) very likely belong to the core genome, while the ‘shell’ set of genes will normally correspond to frequently co-occurring genes. PPanGGOLiN estimates a total of 1537 gene clusters to be a part of the ‘shell’ category for the *Prochlorococcus_A* gene set. For the same gene set, mOTUpan estimates 1637 gene clusters to be part of the core genome. The core estimate of mOTUpan seems to be close to the sum of ‘persistent’ and ‘shell’ (1853 gene clusters). The three closed genomes have 1883 gene clusters, making the ‘persistent + shell’ estimate probably an overestimate of the core genome. The ‘shell’ set of gene clusters is picking up genes that are probably not all from the core but rather frequently occurring accessory operons. This is shown in the heatmap in Figure 4. The gene clusters, which mOTUpan called accessory and PPanGGOLiN called shell, seem to belong to blocks of gene clusters absent in sets of highly complete genomes, hinting at very prevalent operons of accessory genes. Conversely, gene clusters in mOTUpan’s accessory and PPanGGOLiN’s shell seem to be very prevalent gene clusters that have only a diffuse pattern hinting at single mobile genes, for example. This analysis also shows the robustness of mOTUpan to estimate the true core genome from more noisy mOTUs.

Calculations of the core genome using mOTUpan with the 3 closed genomes and 16 genomes with completeness >95% of the *Prochlorococcus_A* cluster estimate 1644 gene clusters in the core (1714 ‘persistent’ gene clusters with PPanGGOLiN). This is probably an upper bound to the size of the core of this *Prochlorococcus_A* mOTU, as additional microdiversity and noise would only remove genes from this, making the 1637 gene clusters predicted to make up the core in mOTUpan for the full set a better estimate than either PPanGGOLiN’s ‘shell’ set (316 clusters) or ‘persistent + shell’ set (1853 clusters).

This generally shows that mOTUpan can predict a core genome very similarly to other state-of-the-art tools, while at the same time being more robust over broader ranges of genome completeness in comparison to those tools.

In order to get an idea on the effect of completeness on the core-genome estimation using mOTUpan, we generated data for 10000 idealized mOTUpan runs. For each run, one ‘good’ genome (a random genome of completeness >45%, picked randomly) and a variable number of ‘bad’ genomes (of completeness <45%) were picked. Empirical true and false positive rates were computed as in Supplementary Figure S1 to evaluate performance in these hard border cases. The completeness of the ‘good’ genome controls mainly the amount of core genes that can be retrieved (Supplementary Figure S2A), and an excess of ‘bad’ genomes seems to reduce the number of core genes retrieved (Supplementary Figure S2B). However, increased number of ‘bad’ genomes added can decrease false positive rate (Supplementary Figure S2C). Also, there is a large amount of noise around the quality of the prediction; this makes selecting a good set of genomes and parameters complicated. The bootstrapping false positive rate can be of help as it seems to be a good predictor for the true positive rate (Supplementary Figures S1B and S2D).

Additionally, to show the effect of genome completeness on the core-genome estimation we ran mOTUpan 30 times for random subsets of 100 genomes of the *Prochlorococcus_A* mOTU with estimated completeness in the range of 0–50%, 50–70% and 70–100% (Supplementary Figure S3). By removing genomes with higher completeness value in the tested subset, mOTUpan expectedly recovers a lower fraction of the core genome.

mOTUpan can be used in a number of ways. It can obviously be used to study pan-genome structure at large scale and with noisier data. This comes with some caveats; i.e. the method is highly dependent on the gene-clustering method used and it is very hard to evaluate the correctness of these at a larger scale. Additionally, mOTUpan can only classify genes that actually are in the genomes that are analyzed. Accordingly, genes that are hard to assemble or bin (due to different *k*-mer or abundance profiles) will be overlooked, leading to an inevitable underestimate of the accessory genomes. Another known issue is that uneven representations of subclades in a genome set might lead to the core of the dominating subclade to be computed. This, however, is easily spotted by a strong decrease of posterior completeness estimates and mOTUpan will print a warning for these cases. Additionally, initial estimation of completeness could potentially impact the core size calculation by mOTUpan. For those novel taxa that are poorly characterized, we might have an overestimation of completeness for the genomes, which might affect the mOTUpan core size calculation. These effects can be evaluated by the bootstrapping method. As shown in Supplementary Figure S1, the false positive rate computed with the bootstrapping method relates well to the accuracy of the core calculation and should single out if the inputted combination of the bins is problematic. It is to be noted though that the estimated false positive rates are conservative (see Supplementary Figure S1). It has to be noted that in the absence of higher quality genomes in an mOTU, estimates of core genomes will be accurate, but might be very partial. However, using the bootstrapped false positive rates allows us to easily detect problematic cases. Nevertheless, it is the only tool available that can do this type of analysis, and should hence be an invaluable resource for biodiversity exploration and comparative genomics. While PPanGGOLiN is performing very well with noisy data, the specific purpose and scope of this tool are different. PPanGGOLiN can be leveraged if one needs to select and identify core genes to, for example, make a core phylogeny, and mOTUpan is a reliable choice for estimating and exploring the core and/or accessory genome structure. Another important use envisioned for mOTUpan is to strengthen functional predictions for metagenomic projects. Rather than relying on single MAGs where the presence of specific genes can be questioned, mOTUpan can robustly quantify this presence as long as highly similar MAGs are available (which is often the case in medium- to large-scale metagenomic project). Notably, it can be used with a variety of genome-encoded traits, and the currently available version has parsers available for Roary, PPanGGOLiN, eggNOG-mapper (21), mmseqs2 (20) and anvi’o (22), with possibly more to be included later.

Ultimately, mOTUpan introduces and enables a new type of analysis within the field of microbial genomics, i.e. the usage of presence–absence of genome-encoded traits combined with some Bayesian computation to predict gene content in a genome set. This approach can be expanded into a number of different directions. We can, for example, move from presence–absence to gene count, or use this approach for gene-linkage assessment to estimate whether some traits co-occur more often than by chance.

## DATA AND CODE AVAILABILITY

The mOTUpan software is written in Python 3 and is freely available under GPL 3.0 license via GitHub in the mOTUlizer package at github.com/moritzbuck/mOTUlizer. A conda recipe and pip package for user-friendly installation are also available in the appropriate repository. Scripts used for the analyses in this paper can be found at github.com/moritzbuck/mOTUlizer/tree/master/mOTUlizer/scripts. The data used for benchmarking are from the GTDB (3) (release 95), available at gtdb.ecogenomic.org (with actual genomes at RefSeq and GenBank); GORG-Tropics (19), available under GenBank at PRJEB33281; and the StratFreshDB (17).

## Supplementary Material

lqac060_Supplemental_Files
