# Supplementary material for: mOTUpan: a robust Bayesian approach to leverage metagenome-assembled genomes for core-genome estimation
Source: NAR Genom Bioinform. 2022 Aug 15;4(3):lqac060. doi: 10.1093/nargab/lqac060 (PMC9376867; doi:10.1093/nargab/lqac060)
Supplement: lqac060_Supplemental_Files [file lqac060_supplemental_files.zip › Buck_et_al_-_Manuscript_v4_-_supplemental_figures.pdf]

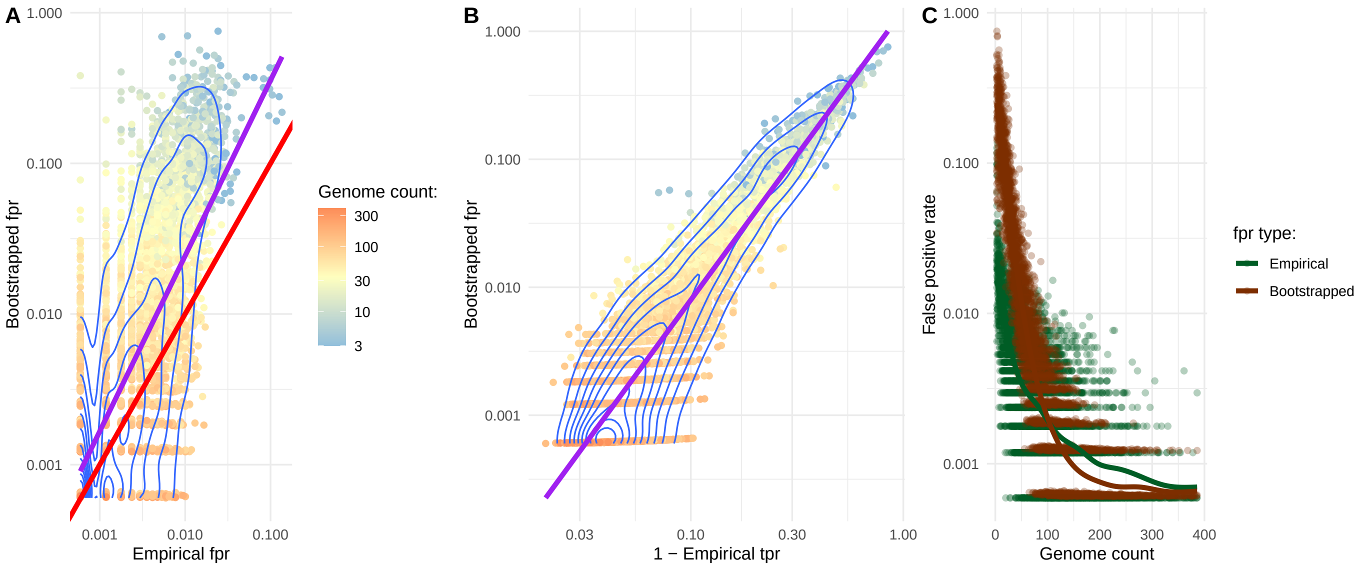

Supplementary Figure S 1: Bootstrapped false positive rate evaluation of the mOTUpan core estimations. mOTUpan runs from Figure 2 were used for this analysis. A) Bootstrapped false positive rate versus the empirical false positive rate. Purple line represents the linear regression ($r^2 = 0.4982$), red: the line where both estimates are equal. B) Bootstrapped true positive rate versus the empirical false positive rate. Purple line represents the linear regression ($r^2 = 0.8495$). C) the value of false positive rate versus number of genomes included in the mOTUpan run. Local polynomial regression fitting is used in the panel C. Data available in supplemental table S3.

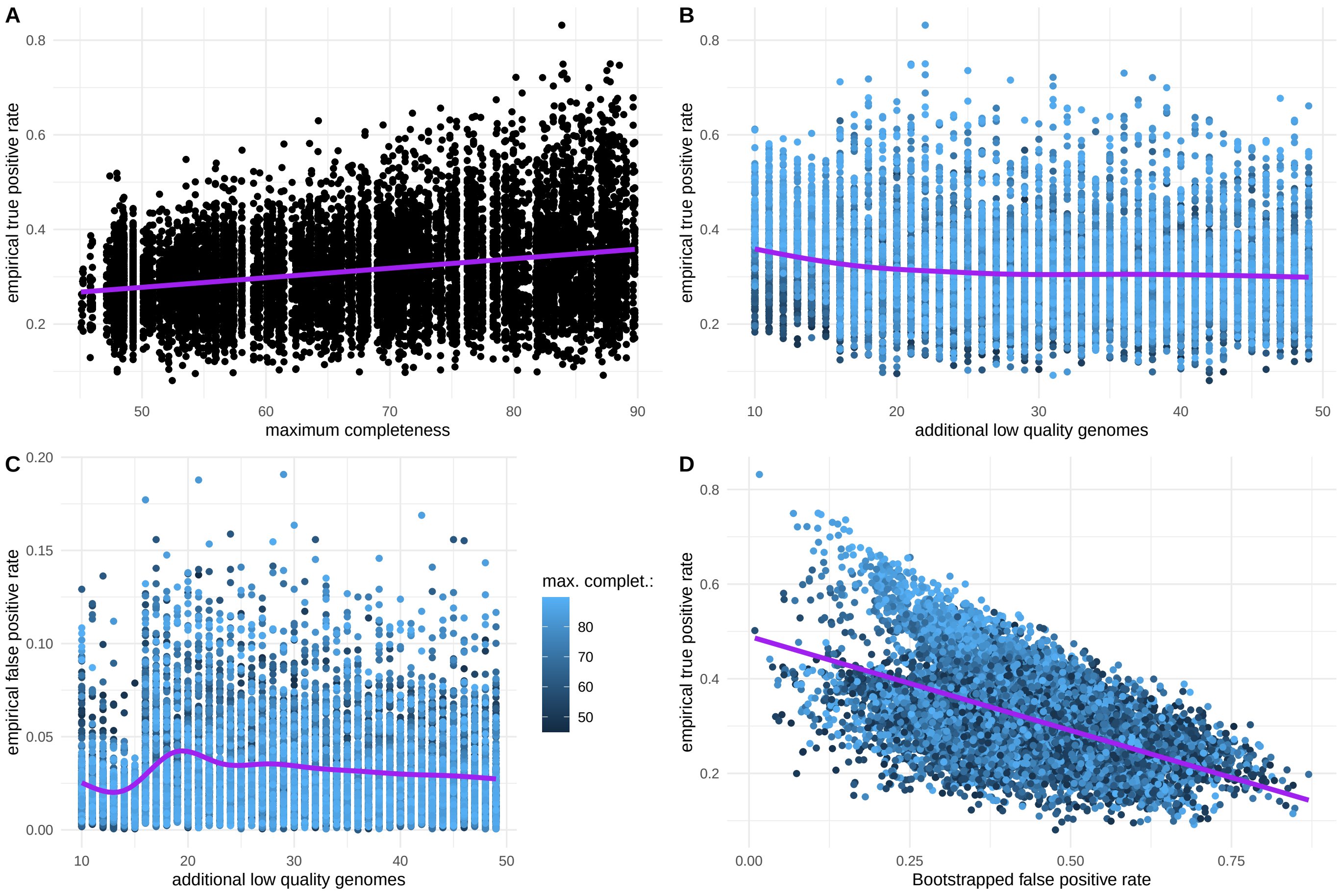

Supplementary Figure S 2: Effect of low quality genomes. mOTUpan was ran 10.000 times over random subsets of the Prochlorococcus_A species, for each subset one “good” genome (a random genome of completeness over 45%, picked uniformly) and a variable number of “bad” genomes (of completeness below 45%) where picked randomly. Empirical true and false positive rates were computed as in Supplementary Figure S1. Purple line on panel A and D are linear regressions ($r^2 = 0.07539$ and $r^2 = 0.2974$ respectively), on panel B and C local polynomial regression fitting is used. Data available in supplemental table S4.

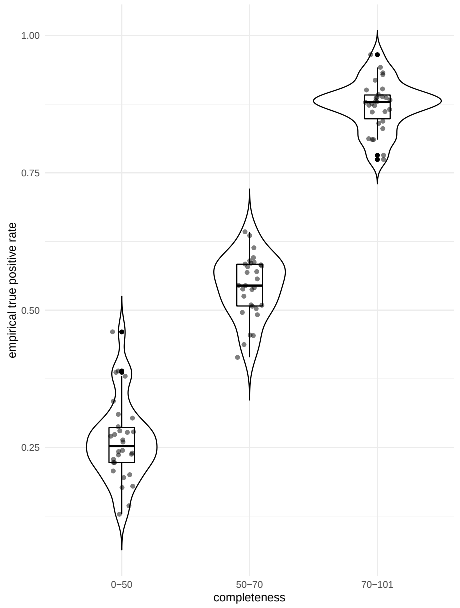

Supplementary Figure S 3: General effect of genome completeness on the estimated fraction of the core genome. For 3 completeness ranges (0-50%, 50-70% and 70-100%), mOTUpan was ran 30 times for random subsets of 100 genomes of the Prochlorococcus_A species belonging to that completeness range. Empirical true positive rates were computed as in Supplementary Figure S1. Data available in supplemental table S5.
